# Rationale and development of an on-line quality assurance programme for colposcopy in a population-based cervical screening setting in Italy

**DOI:** 10.1186/1472-6963-13-237

**Published:** 2013-06-28

**Authors:** Lauro Bucchi, Paolo Cristiani, Silvano Costa, Patrizia Schincaglia, Paola Garutti, Priscilla Sassoli de Bianchi, Carlo Naldoni, Oswaldo Olea, Mario Sideri

**Affiliations:** 1 Romagna Cancer Registry, IRST, 47014 Meldola, Forlì, Italy; 2 Cervical Cancer Screening Unit, Bologna Health Care District, Bologna, Italy; 3 Department of Obstetrics and Gynaecology, St. Orsola Hospital, University of Bologna, Bologna, Italy; 4 Cancer Prevention Centre, Ravenna Health Care District, Ravenna, Italy; 5 Department of Obstetrics and Ginaecology, St. Anna Hospital, University of Ferrara, Ferrara, Italy; 6 Department of Health, Regione Emilia-Romagna, Bologna, Italy; 7 Applicazioni-web.info, Firenze, Italy; 8 Preventive Gynaecology Unit, European Institute of Oncology, Milan, Italy

**Keywords:** Quality Assurance, Colposcopy, Cervical Cancer Screening, Interobserver Agreement, Internet, Digital

## Abstract

**Background:**

Colposcopy, the key step in the management of women with abnormal Pap smear results, is a visual technique prone to observer variation, which implies the need for prolonged apprenticeship, continuous training, and quality assurance (QA) measures. Colposcopy QA programmes vary in level of responsibility of organizing subjects, geographic coverage, scope, model, and type of actions. The programmes addressing the clinical standards of colposcopy (quality of examination and appropriateness of clinical decisions) are more limited in space and less sustainable over time than those focused on the provision of the service (resources, accessibility, etc.). This article reports on the protocol of a QA programme targeting the clinical quality of colposcopy in a population-based cervical screening service in an administrative region of northern Italy.

**Methods/design:**

After a situation analysis of local colposcopy audit practices and previous QA initiatives, a permanent web-based QA programme was developed. The design places more emphasis on providing education and feedback to participants than on testing them. The technical core is a log-in web application accessible on the website of the regional Administration. The primary objectives are to provide (1) a practical opportunity for retraining of screening colposcopists, and (2) a platform for them to interact with colposcopists from other settings and regions through exchange and discussion of digital colposcopic images. The retraining function is based on repeated QA sessions in which the registered colposcopists log-in, classify a posted set of colpophotographs, and receive on line a set of personal feedback data. Each session ends with a plenary seminar featuring the presentation of overall results and an interactive review of the test set of colpophotographs. This is meant to be a forum for an open exchange of views that may lead to more knowledge and more diagnostic homogeneity. The protocol includes the criteria for selection of colpophotographs and the rationale for colposcopic gold standards.

**Discussion:**

This programme is an ongoing initiative open to further developments, in particular in the area of basic training. It uses the infrastructure of the internet to give a novel solution to technical problems affecting colposcopy QA in population-based screening services.

## Background

### Introduction

#### (a) Colposcopy

Colposcopy, a visual examination of the uterine cervix and vagina using a lighted field microscope after the application of a diluted solution of acetic acid and Lugol’s iodine solution as staining agents, is the key step in the management of women with abnormal Pap smear results. Colposcopy aims at detecting macroscopic changes in tissue features such as colour and morphology. Comparison of these features with established patterns of disease enables the clinician to classify the lesions and to identify abnormal areas that may need to be biopsied in order to detect precancerous or cancerous lesions [1].

However, the colposcopic impression of any abnormality is prone to observer variation. This is potentially associated with low intra- and interobserver agreement in identifying cervical lesions, and high biopsy sampling error rate. An important study has raised concern that errors in the selection of biopsy site may lead to low sensitivity for detection of high-grade cervical intraepithelial neoplasia (CIN) [2]. The importance of these problems is heightened by the increasing use of human papillomavirus virus (HPV) testing for cervical cancer screening. Because HPV test has high sensitivity but limited specificity, the role of colposcopy in the identification of patients with clinically significant lesions becomes even more crucial [3].

To cope with these problems, some novel approaches have been suggested including the identification of a limited set of well-defined and reproducible colposcopic features of cervical abnormalities, the development of computer-assisted diagnosis, the collection of additional biopsy specimens from the worst-looking lesion and other abnormal areas, and the collection of random biopsy specimens from all cervical quadrants [3].

At present, however, the diagnostic performance of a colposcopist depends solely on his visual skills and, thus, on his experience and competence in comparing the perceived pattern with established features of premalignant and malignant lesions [1]. As a consequence, colposcopy requires a period of apprenticeship, continuous formal training, sufficient workload and, more important, auditing and quality assurance (QA) measures.

In this article, we report the protocol of a colposcopy QA programme that is underway in a population-based cervical screening setting in the Emilia-Romagna Region of northern Italy.

#### (b) Study setting

The Emilia-Romagna Region, one of the largest administrative regions of Italy, is currently subdivided into 11 health care districts. General characteristics of cervical screening service in the area are described elsewhere [4-6]. In brief, every three years since 1997, resident women aged 25–64 years (*n* = 1,248,000 on 1 January 2012) are invited by a personal letter to attend for a free Pap smear. Current response rate is 58% [7]. Pap smears are taken by midwives at the district screening centres. Abnormal Pap smear results are notified to women by telephone. Colposcopy assessment is carried out free of charge by gynaecologists and gynecologist-oncologists at selected clinics. These follow guidelines from the regional Department of Health [8].

Routine audit and QA initiatives are under the responsibility of the regional Department of Health. Since 2006, an annual statistical audit has been done according to guidelines from the National Centre for Screening Monitoring (Italian: *Osservatorio Nazionale Screening*) [7]. QA initiatives are designed by specially appointed steering committees composed of specialists from the screening centres and the public hospitals [5,6].

In 2009, the Steering Committee for Clinical QA in Cervical Screening (hereby referred to as ‘the Committee’), of which we are current members, received the mandate to develop a QA programme for colposcopy clinics. The programme protocol was written in a simplified manner to help potential participants during the recruitment process. The current paper presents an expanded and partly updated version, which was written after full implementation of the programme in the form of a retrospective article.

### Conceptualisation of colposcopy QA

The Committee performed a review of the literature in order to establish a conceptual framework for the planning and design of the programme.

#### (a) Basic perspective

The numerous terms used to describe the quality in health care as well as the tools used to improve it vary between countries, between stakeholders, and over time [9]. Since there is no international classification of these concepts, the terms are often used interchangeably although there can be differences in meaning. In Italy, this is the case for ‘colposcopy audit’ and ‘colposcopy QA’. These terms, in fact, indicate different perspectives.

##### Colposcopy audit

The objectives of colposcopy audit activities are to review and evaluate the adequacy and resources of colposcopy clinics, the quality of colposcopic examination, and the appropriateness of clinical decisions [10]. They use several different techniques including, for example: (1) assessment of the degree of adherence of colposcopy clinics to standards of practice [11-13]; (2) evaluation of survey data from colposcopy clinics on the management of defined categories of cervical lesions [14]; (3) random or systematic retrospective review of colposcopic impressions reported in case notes with assessment of their correlation with cytological and histological diagnosis [15-18]; (4) review of colposcopy records of patients diagnosed with cervical cancer [19]; (5) collection of information by focus groups of patients or by questionnaire; and (6) analysis of complaints data [10].

##### Colposcopy QA

Colposcopy audit can be designed to stand alone or be a part of a QA scheme. In fact, the definition of standards and the measurement of their achievement are the basis for a QA scheme. However, the challenge for a colposcopy QA team is not only to identify the areas of colposcopy practice most susceptible of improvement but to target them with appropriate actions. The central component of a QA programme is the development of techniques capable of improving the performance of colposcopy [10].

Often, QA is also identified with the process of accreditation. There is a growing realisation that these two concepts should be kept separate. QA focuses on support for health services in the development of quality improvement processes.

#### (b) Level of responsibility

Colposcopy QA can be carried out by health authorities, public health agencies, scientific organisations, professional bodies, spontaneous groups, or individuals who systematically or occasionally attempt to review and improve their work or that of their teams in a rational manner [10]. The institutional level of responsibility of the individual organiser or organising centre has several implications for the planning of the programme, in particular for the geographical coverage and the degree of continuity in the long term [20].

#### (c) Scope

The scope of colposcopy QA activities may focus on either or both of two main areas, namely:

• the provision of the service (for example: resources, infrastructure, accessibility, and adequacy indicators such as the proportion of patients with recording of the reason for referral); and

• the clinical standards of the service and the appropriateness of clinical decisions.

#### (d) Model

There are some paradigmatic models of colposcopy QA.

##### Agreement studies

Obtaining data on the level of diagnostic agreement on the interpretation of colpophotographs [21] as well as of their digital counterparts [22] is useful for both audit and QA purposes. Agreement coefficients can be computed for a group of observers as a whole, for each observer, and for specific colposcopic patterns. Agreement studies are often associated with intensive courses in the form of interactive slide seminars of study cases [21].

##### Multidisciplinary colposcopy meetings

While most designs for colposcopy QA, including agreement studies, involve the re-evaluation of selected archival material and rely on education, multidisciplinary colposcopy meetings are a real-time approach [23,24] that is directly aimed at correcting certain diagnostic errors. According to the guidelines of the National Health Service cervical screening programme [25], multidisciplinary meetings should focus on cases with significant mismatch between colposcopy, cytopathology, and histopathology. The identification of patients with false-positive and false-negative diagnoses permits avoidance of over-treatment and safe treatment of high-grade precancerous lesions. To this end, multidisciplinary meetings should be held often enough to allow for timely care of patients.

##### On-site colposcopy QA visits

A remarkable example of on-site colposcopy QA visits can be found in the Irish cervical screening programme [26]. The process, which is periodically repeated, begins with a questionnaire survey of colposcopy clinics. As a second phase, a specialist team visits each clinic to perform an on-site audit of the levels of provision of the service. The visit focuses on staffing, infrastructure, communication, continuing medical education, fail-safe procedures, written protocols, waiting times, and diagnostic indicators. After the visit, a written report is provided to the colposcopy staff and the hospital management to identify priority needs.

In Italy, the National Centre for Disease Prevention and Control (Italian: *Centro Nazionale per la Prevenzione e il Controllo delle Malattie*) has launched a pilot project for on-site visits designed for those screening centres in which efficient monitoring systems have not been set up [27].

#### (e) Action

Potential colposcopy QA interventions vary in relation to the objectives pursued. In particular:

• insufficient levels of resources may require administrative decisions;

• adequacy indicators can be improved through technical actions, although feedback of audit information alone may be sufficient to bring about the desired correction;

• failures to achieve clinical standards warrant more structured and direct approaches, such as the professional retraining of colposcopists.

#### (f) Interrelated characteristics of QA programmes

Most of the above aspects are interrelated. The largest colposcopy QA programmes, such as those implemented by health authorities, are generally focused on the provision of the service (that is, infrastructure, accessibility, etc.) rather than on its clinical standards. For example, this is the case for on-site QA visit programmes. In general, these approaches do not involve direct assessment of the physician’s ability to perform a colposcopic examination. Indirect approaches, such as the comparison of the reported colposcopic impression vs. final histological diagnosis [15-18], are more common.

Agreement studies are more colposcopist-oriented. They are mainly devoted to improve the quality of colposcopic examination and the appropriateness of clinical decisions. On the other hand, they are seldom conducted on a regional [28] or community [21] basis. They generally target undefined samples [29,30] or selected groups [31,32] of gynaecologists. Most of these considerations also apply to multidisciplinary colposcopy meetings.

Limitations in geographical coverage of agreement studies and multidisciplinary colposcopy meetings are coupled with problems of continuity in the long term. These schemes are more often introduced on a voluntary basis than through legislation, and are more susceptible to closure or interruption [20].

In summary, QA programmes targeting the clinical standards of colposcopy practice are more limited in space and time than those focused on the provision of the service. This is a serious problem, because the ultimate rationale of an organised cervical screening service is to offer standardised procedures of controlled and assured technical quality to a vast population. Variations in the clinical quality of colposcopy between centres may hamper the effectiveness of screening and cause health inequalities. As a consequence, colposcopy QA programmes should aim at improving both the average clinical level and the homogeneity of the diagnostic performance. Both tasks requires the development of mid/large scale initiatives.

## Methods and design

### Situation analysis

In order to have an overview of the situation in which the QA programme was to take place, the Committee gathered the available information relating to the patterns of provision and the clinical quality of colposcopy in the local screening service. Two main sources of information were identified.

#### (a) Colposcopy audit data

Colposcopy audit data were obtained from the archives of the regional Department of Health. Up to 2005, the main performance indicators of the screening service, including some indicators of colposcopy assessment for women with abnormal Pap smear results, were monitored using a standard set of aggregate data annually collected by the district screening units. In 2006, the electronic datasheet developed by the National Centre for Screening Monitoring for the annual national survey of local programmes was adopted. In the most recent data (Additional file 1), the regional average level of quality of colposcopy service was acceptable, but with considerable variation between health care districts. The proportion of patients with CIN3 and carcinoma classified into the two highest categories of colposcopic impression was among the most fluctuating indicators.

#### (b) Previous QA initiatives

Between 2000 and 2003, the previous Committee implemented some on-site QA initiatives targeting the screening colposcopists [33]. In 2000, a two-day course was held that covered the distinction of normal from abnormal transformation zone, the grading of colposcopic abnormalities (see below: Colposcopic variables), and the identification of invasive cancer. A teaching set of 480 slides obtained with a standard cervicograph was used. Before and after the course, 50 test slides were projected onto a screen, and classified for colposcopic impression, visibility of the squamocolumnar junction (SCJ), and indication for biopsy. In a subsequent seminar, the before-after variation in interobserver agreement was presented and the most controversial images were discussed in an interactive learning session. This programme was repeated in 2001.

In 2002, copies of a newly selected set of 50 slides were sent to all district screening units, and were classified by local colposcopists according to the above variables. A concluding plenary seminar was held.

In 2003, each colposcopist received a CD-Rom containing a specifically designed software package and a new set of 50 digital colposcopic images, which were classified in a fixed time. Responses were returned via e-mail. As in previous QA rounds, a plenary seminar was held to review the results and the most problematic images.

The Committee’s opinion on these initiatives was that they were difficult to set up, expensive, time consuming, and inaccessible to external review. Conversely, their design was appropriate for assessing and improving the colposcopists’ competence.

From the findings of this situation analysis, the Committee concluded that:

• there were sufficient justifications for implementing a QA programme in colposcopy;

• the programme had to use the communication infrastructure of the internet, be implemented on a permanent basis, and be more efficient than previous residential QA initiatives;

• the annual statistical audit of the screening service was sufficient to cover the essential aspects of colposcopy assessment; and

• the variation of the indicators of the diagnostic process across health care districts was large. Reducing this variation was a priority objective.

### Current technical level of digital colposcopy imaging

Since colposcopy is an in vivo procedure, there are objective difficulties in comparing the results *a posteriori* with other examiners as well as a QA team [21]. The problems can be summarized into two broad categories: (1) it is difficult to obtain highly representative still images and videos that can allow the colposcopic examination to be quality-assured by other observers; and (2) conventional binocular colposcopes do not provide neither guidance for biopsy nor documentation of the biopsy procedure.

For years, studies of interobserver variation have used colpophotographs (also referred to as cervigrams). The colpophotograph is a 35-mm picture of the cervix taken under magnification during colposcopy approximately one minute after acetic acid application.

With the introduction of digital video colposcopes, the need to project images has been eliminated, since an observer can view them on the computer screen. Compared with colpophotographs, digital colposcopic images suffer from a lower level of standardization. In general, however, the diagnostic reliability of digital colposcopy compared with conventional colposcopy is considered acceptable [1]. Particularly if images are free from artifacts and imperfections, digital colposcopy can be used for the visual assessment of cervical abnormalities.

The introduction of the internet and e-mail communications has greatly facilitated the review of digital colposcopic images outside the examination room. There are two main types of on-line transmission of digitalized images [1]. In the ‘computer-based’ transmission, images are first stored using a computer software and then forwarded, whereas the ‘network-based’ transmission uses an infrastructure composed of a technologically advanced hardware and rapid telecommunication lines. The latter system has only a slightly greater sensitivity for the detection of high-grade cervical lesions [34,35].

Based on current knowledge [1], the Committee considered the technical level of digital colposcopy imaging to be acceptable for the purposes of a web-based colposcopy QA programme.

### Definition and objectives

The Committee developed the programme. The technical basis consists of an easy-to-handle software that is permanently made available at a log-in web application. The objectives of the programme are to provide:

• a practical opportunity for permanent retraining of the colposcopists’ staff of the regional screening service; and

• a platform for them to load and exchange their own digital colposcopic images, to present and obtain feedback on their colposcopic impressions, and to interact with colposcopists from different settings and geographical regions.

The programme has not administrative functions. It is not designed to: set up a monitoring system for purposes of central control; rank the colposcopists; certify their competence; collect single screening centre’s data for accreditation purposes; and provide data to national screening authorities. The programme is colposcopist-oriented, and places more emphasis on providing education and feedback to colposcopists than on testing them.

The objectives and methods of the programme (including the use of personal information and results) were publicly announced. The details were explained during a preparatory meeting in late 2010.

### Eligibility

Participation is open to all colposcopists involved in secondary prevention of cervical cancer at any level. Eligibility extends to professionals from outside the regional area as well as those working in the clinical setting and the private sector. Access to the application is free of charge for all users.

The idea that all professionals who are interested should be given the opportunity to participate reflects the increasing view that opportunistic screening should be integrated into organised screening [36]. In all geographical areas targeted by organised screening systems, a substantial volume of opportunistic screening co-exists. It is generally agreed upon that organised screening could be improved by extending evaluation procedures to include opportunistic activity [36]. Central to this task are the creation of population-based databases and the obligatory registration of all screening Pap smears. By analogy, a web-based platform for colposcopy QA can be used as a tool to extend the concept of integration of opportunistic screening from the basic level to colposcopy assessment.

### Design

The programme has a two-phase design.

#### (a) First QA session

In this baseline phase, which was carried out in early 2011, the programme was targeted at the staff of colposcopists working at that time in the screening centres of the region. Participation was on a voluntary basis but was strongly advised.

The web application was implemented and made accessible at the website of the regional Administration of Emilia-Romagna. After obligatory registration, the colposcopists logged-in with a user name and a password to the web application, and underwent a test of their diagnostic performance.

They viewed a posted set of 50 colpophotographs selected by the Committee and they classified each according to the following variables: (1) colposcopic impression; (2) visibility of the SCJ; (3) need for biopsy; and (4) most appropriate biopsy site. The participants were mutually unaware of their reports. They compiled this information using an on-line reporting form and immediately received a set of individual feedback data.

Table 1 shows the classification scheme. The variable ‘colposcopic impression’ was classified into four categories: negative; abnormal, grade 1; abnormal, grade 2; and suspected invasive cancer. Despite the simplified terminology, these categories were equivalent to those of the International Federation for Cervical Pathology and Colposcopy (IFCPC) classification of 2002, namely: normal colposcopic findings; abnormal colposcopic findings, minor changes; abnormal colposcopic findings, major changes; and colposcopic features suggestive of invasive cancer [37]. Four colpophotographs representing these categories are shown in the Additional file 2.

**Table 1:** Colposcopic variables for the quality assurance programme

**Variable**	**Categories**
Colposcopic impression*	Negative
	Abnormal, grade 1
	Abnormal, grade 2
	Suspected invasive cancer
Squamocolumnar junction	Visible
	Not or not entirely visible
Indication for biopsy	Yes
	No
Biopsy site†	Correct
	Incorrect

* The four categories of the variable ‘colposcopic impression’ were equivalent to those of the International Federation for Cervical Pathology and Colposcopy (IFCPC) classification of 2002, namely: normal colposcopic findings; abnormal colposcopic findings, minor changes; abnormal colposcopic findings, major changes; and colposcopic features suggestive of invasive cancer [37].

† Correctness of biopsy site according to the programme Steering Committee's judgment.

The first QA session ended with a colposcopy seminar. The protocol details presented hereafter concern the fist QA session.

#### (b) Prospective activities

After this start-up phase, the programme will continue along three main lines. Their planning is to be considered a work in progress, which will be updated as implementation progresses and results are achieved.

##### Repeated QA sessions for screening colposcopists

All regional QA sessions that will be held in the future will have the same general format as the first session. The criteria for selection of cases, however, are open to modifications. For example, if a good level of diagnostic accuracy for the most common colposcopic presentations will be reached, images showing uncommon or rare colposcopic patterns could be included in the test set.

##### Enrollment of colposcopists from other settings and other Italian regions

This will offer the opportunity for a comparative approach to analysis of our data. To this end, however, a properly designed study needs to be undertaken, possibly under the auspices of a national screening authority. In particular, appropriate methods will have to be developed in order to ensure the participation of an unselected sample of Italian screening colposcopists.

##### Permanent availability of a platform for exchange and discussion of digital colposcopic images

The colpophotographs loaded by users will be evaluated by the Committee with an empirical approach, along with their potential uses. This material would enlarge the spectrum of colposcopy patterns typical of routine practice, although its suitability for use in QA sessions would need to be assessed.

### Concluding colposcopy seminar

The concluding event – and a major component – of the first QA session was a colposcopy seminar. This will also be the case for all future QA sessions designed for the personnel staffing the screening centres. A colposcopy seminar is an intensive course held by the Committee which includes (1) the personal communication of individual test results to participants; (2) a statistical presentation of overall test results; and (3) an interactive review of slides of all digital colpophotographs. A seminar normally lasts 5 hours.

#### (a) Personal communication of individual test results

Each participant receives a personal report showing his data for agreement with the Committee on the colposcopic impression, visibility of the SCJ, need for biopsy, and selection of biopsy site.

#### (b) Presentation of overall test results

The plenary session begins with the presentation of overall (group) results including total values, distributions, and ranges of agreement percentages and kappa agreement coefficients for colposcopic impression, visibility of the SCJ, need for biopsy, and selection of biopsy site.

#### (c) Interactive review of slides

The rationale of joint review of slides is that a professional interaction between the Committee and the participants may expand the educational effect of individual participation to the test. Slides are projected on a screen in a darkened hall. Each slide is accompanied by information regarding the colposcopic impression formulated by the Committee and by the majority of participants, the proportion of participants who agreed with the Committee on the need for biopsy and on the most appropriate site, and the most severe histological diagnosis.

Participants of the QA session are encouraged to discuss the discrepancies between their evaluation and that by the Committee. An independent external expert takes part in the discussion, contributing complementary knowledge and experience. The interactive review is meant to be a discussion between peers. The Committee's classification is primarily provided with the objective to make the discussion more focused and straightforward. The ultimate purpose is to offer a forum for an open exchange of views that may lead to more knowledge and more diagnostic homogeneity.

### Web application

The web application in which the preregistered participants register, log-in, and access the test set of images through the internet (https://sanita.regione.emilia-romagna.it/colposcopia) is the technical core of the programme. Details of the programming languages and the software programme used to create the application, its structure and design, and a description of pre-registration, registration, and log-in functions can be found in the Additional file 3.

### Selection of the test set of colpophotographs

#### (a) Acquisition of images

Two of us (PS and PG) selected a basic set of 250 high-definition digital colpophotographs from the screening centres of the two health care districts. Technical details of acquisition of images are described in the Additional file 4.

#### (b) Criteria

From the initial set of images, four of us (PC, PS, PG, and SC) selected 50 images with the following characteristics: (1) they were well-representative of the four categories of the IFCPC classification of 2002 [37] (Table 1); (2) they were of high technical quality; (3) the cervix was entirely visible; (4) there was no visible excess mucus accumulation; (5) there were no light reflections or colour artifacts or shaded areas; (6) the patient had not been treated previously; and (7) information on patient age, last screening Pap smear report, and HPV test result was available. Original histological information, including normal histology and biopsy not performed, was known to the selectors but was not assumed to represent a gold standard for the colposcopic impression.

The rationale for criteria 1–5 was two-fold: first, a colposcopy QA programme should target the basic presentations of normal and affected tissues of the cervix with which to compare the spectrum of colposcopic patterns encountered in practice; and, second, colposcopic images used in a QA setting should be as least controversial as possible. Controversies weaken the organisers’ credibility, and decrease the educational effect of the programme.

#### (c) Validity

Using high-quality images of classic colposcopic patterns leads to an overestimate of agreement and accuracy measures. In general, it is a requirement for external QA schemes to simulate the daily practice conditions as closely as possible. As far the current programme is concerned, however, this is a purely theoretical argument, since we do not aim at interregional comparisons with results obtained elsewhere using different test sets, and the data on the colposcopists’ performance are not used for administrative purposes.

Furthermore, examination of a test set of colposcopic images is always and inevitably impossible to compare with normal practice, because the test environment is totally artificial and the test set includes a disproportionately high proportion of abnormal colposcopic findings. As a consequence, inclusion of imperfect images could not significantly improve the comparability of the programme with the daily practice conditions.

These limitations suggest that agreement measures on colposcopic variables have no absolute significance, and should only be used for internal comparisons for a given test environment (for example: comparisons between groups of colposcopists defined by the number of years of experience, etc.).

### Colposcopic variables

The colposcopic variables according to which to classify the test set of 50 colpophotographs were selected based on the following rationale.

#### (a) Colposcopic impression

The primary responsibility of the colposcopist is to rule out the presence of invasive cancer. This involves the identification of the basic signs of invasion. To assess the presence and grade of a lesion, an appropriate recognition of the entire transformation zone should be carried out. The aim of colposcopy is to provide an objective guide to differentiate between normal, minimally abnormal and significantly abnormal colposcopic findings, in order to select the site for biopsy. After acetic acid application, the margins, colour tones, vascular pattern, and surface contour are graded into minor changes (or abnormal, grade 1) suggesting low-grade CIN and major changes (or abnormal, grade 2) indicating high-grade CIN.

#### (b) Visibility of the SCJ

The second key objective of colposcopic examination is to assess the topographic distribution of the lesion, since the endocervical dislocation of the abnormal transformation zone occurs frequently. The colposcopist should be able to determine the visibility of the SCJ in order to plan further diagnostic and treatment procedures. Accurate recognition of the SCJ has a major clinical impact, since it yields useful information in tailoring the excisional treatment.

#### (c, d) Need for biopsy and biopsy site

Identifying the areas of the cervix from which a biopsy would be obtained is the key in order to rule out an invasive disease. In fact, colposcopy is subject to high biopsy sampling error rate [30]. The web application allows the user to choose only one biopsy site. In a real world situation, conversely, obtaining multiple biopsies from large or multifocal lesions is a common approach. In a clinical setting, moreover, the histological diagnosis of biopsy specimens should be considered in context with the colposcopic impression, the Pap smear report, and the HPV test result. In case of substantial discrepancy, a loop electrosurgical excision procedure may be performed. This confirms that the overall sensitivity of the diagnostic process cannot be directly inferred from the sensitivity of colposcopically guided biopsy in a QA environment.

### Gold standards

The design of data analysis was based on precise assumptions about the question of colposcopic and histological gold standards.

#### (a) Colposcopic gold standard

The fact that colposcopy is a subjective technique means that an objective gold standard for colposcopic impression is lacking, and that a considerable degree of observer variation is inevitable.

This provided the rationale for considering interobserver agreement as a major outcome measure of the QA programme, since: (a) in the absence of a gold standard, interobserver agreement is an acceptable measure of quality of colposcopic examination, especially in a large sample of observers; and (b) reducing interobserver variation to a low level is a priority objective for the screening service.

The Committee’s classification of images according to colposcopic impression and visibility of the SCJ was not considered to formally represent a gold standard, that is, an absolute reference. However, the high level of expertise of Committee members suggested to use their classification as a reference of sufficient quality for the public health purposes of the programme. As a consequence, the comparison between the participants and the Committee had a central role in data analysis, although it was made using agreement statistics, not accuracy measures.

The biopsy sites selected by the colposcopists were automatically classified into correct and incorrect, which was inevitably treated as a gold standard. In the Committee’s classification, the impression of abnormal colposcopic findings translated into an indication for biopsy, and the impression of normal findings into the opposite indication. In a small number of cases, the participating colposcopists did not follow this criterion of equivalence. We assumed that these responses could result from diagnostic uncertainties rather than material errors, and we retained them in analysis.

#### (b) Histological gold standard

The Committee did not use the original histological diagnosis as a gold standard to formulate a colposcopic impression. The colposcopic impression is not a direct predictor of the histological grading of a lesion, since errors in the selection of biopsy site may result in the missing of one quarter of prevalent CIN2 or CIN3 [2], and there is only moderate agreement even between expert pathologists on the interpretation of biopsy and surgical specimens [38].

Despite this, many screening monitoring systems include a cross-tabulation of colposcopic impressions with the most severe histological diagnoses. This is because histological diagnosis can be considered a ‘public health gold standard’. Accuracy of colposcopy towards histological diagnosis provides an approximate measure of the probability for a woman to receive a false-negative or false-positive colposcopy evaluation. For this reason, a cross-tabulation of colposcopic impressions with the most severe histological diagnoses was included among the outcomes of data analysis.

### Analysis and evaluation issues

#### (a) Analysis of test results

Data analysis was mainly based on agreement statistics, i.e., percent observed agreement and Cohen’s kappa coefficient. These measures were used to assess the single colposcopist’s test results as well as the overall results, which were presented at the first colposcopy seminar. Agreement statistics play a prominent role in the development of diagnostic tests. Disagreements for particular diagnostic categories can be used to modify the reporting system and improve the test performance.

A second stage of data analysis was planned for publication purposes. Results on colposcopic impression, decision for biopsy, correctness of biopsy site, and other secondary topics will be reported. Analysis of data on colposcopic impression will include, among others, the evaluation of statistical significance of differences between kappa values, and the assessment of the degree and direction of colposcopist-Committee disagreement. Analysis of data on biopsy will include, among others, a logistic regression analysis of factors associated with a correct decision for biopsy and a correct selection of biopsy site.

#### (b) Evaluation of impact

To prospectively assess the effectiveness of the training session in improving the diagnostic performance of the staff, the correlation between the colposcopic impression and the histological diagnosis will be evaluated using a “before-after” approach, i.e., comparing data for the year 2012 with the years 2006–2011 on a regional basis. Other approaches are impossible at present due to limitations in data availability.

### Ethics and funding considerations

The QA programme described in this protocol did not involve patients or patient data and, according to the Italian legislation, it did not require formal ethical committee approval. The digital colpophotographs used for the programme were kept anonymous. Approval for their use was obtained from the Direction Boards of the screening centres of origin. Staff involved in data analysis had access only to deidentified physician data. The application used for this programme conforms to the technical and security specifications set by the Computer Information Service of the regional Administration of Emilia-Romagna.

The programme did not receive funding or assistance from commercial organisations or external funding bodies. Both direct and indirect costs were entirely paid by the Department of Health of the Emilia-Romagna Region. The funder had no role in the design of the protocol, the preparation of the manuscript, or the decision to submit for publication.

## Discussion

Several innovative digital colposcopy techniques have been developed in the last years [1]. Despite the promising results of some studies, they have not yet been introduced on a large scale at the office level. As a parallel problem, web-based tools have been rarely used for colposcopy QA. Although the combination of digital colposcopy imaging with the communication infrastructure of the web would greatly facilitate the implementation of mid/large scale programmes, there have been very few examples of intercolposcopist agreement study with large and defined geographical basis [28].

The design of this programme was complicated by the lack of a gold standard for colposcopy interpretation, the subjectivity of criteria for selection of the test set of colpophotographs, and the artificiality of the test conditions. These well-known limitations suggest a cautious interpretation of results, but do not constitute sufficient justification for not implementing QA measures for colposcopy practice.

This programme fulfils some important theoretical requirements for an effective QA [10]. With their background and long-standing experience in colposcopy, the Committee members offer a specific professional leadership to participants. The Committee operates on behalf of and with approval from the regional Department of Health and, thus, it works in a supportive environment. Perhaps more important, the methods of the programme and the use of personal information are well-accepted by participants.

In other areas of medical practice, it has been suggested that QA schemes are sustainable over time only with the introduction of registration fees to cover running costs [20]. Our situation differs in that both direct costs (start-up and maintenance) and indirect costs (work hours lost) are paid by the regional Department of Health. Moreover, since the programme involves the loss of fewer work hours compared with residential programmes, initial costs of implementation will be progressively recouped. In brief, continuity in the long term will only depend on adequate turn-over of membership of the Committee.

This programme is open to further developments. In the area of basic training, we are considering the idea of training sessions specifically tailored to the needs of non-experienced colposcopists who are candidates to replace those going to retire. From many points of view, however, the most interesting perspective is to undertake a large interobserver agreement study among Italian colposcopists in which the focus is on the IFCPC classification of 2011 [39].

The first QA session took place between December 2010 and February 2011. The first colposcopy seminar was held in Bologna in May 2011. The interobserver agreement data presented in that meeting, plus a selection of original analyses, will be reported in separate publications.

## Abbreviations

CIN: Cervical intraepithelial neoplasia; HPV: Human papillomavirus virus; IFCPC: International Federation for Cervical Pathology and Colposcopy; QA: Quality assurance; SCJ: Squamocolumnar junction.

## Competing interests

The authors declare that they have no financial and non-financial competing interests.

## Authors’ contributions

LB participated to the development of the protocol and wrote the manuscript. PC, SC, PS, and PG developed the protocol. PSdB designed the procedures for data acquisition. CN supervised the programme. OO created the web application. MS acted as a guarantor for the programme and performed the critical revision of the manuscript. All authors read and approved the final manuscript.

## Pre-publication history

The pre-publication history for this paper can be accessed here:

http://www.biomedcentral.com/1472-6963/13/237/prepub

## Supplementary Material

Additional file 1 **Performance indicators routinely audited in colposcopy clinics of the Emilia-Romagna Region of Italy and observed data.** A Table shows the list of performance indicators routinely audited in the colposcopy clinics of the Emilia-Romagna Region of Italy and, for each, the observed regional average and the range between health care districts. Click here for file

Additional file 2 **Digital colpophotographs representing the four categories of the International Federation for Cervical Pathology and Colposcopy classification of 2002.** Four figures show digital colpophotographs taken with standard technique in one of the screening centres of the Emilia-Romagna Region of Italy, and considered by the programme Steering Committee to be well-representative of the definitions of normal colposcopic findings, abnormal colposcopic findings-minor changes, abnormal colposcopic findings-major changes, and colposcopic features suggestive of invasive cancer (in the programme, these categories were referred to as: negative; abnormal, grade 1; abnormal, grade 2; and suspected invasive cancer). Click here for file

Additional file 3 **Technical details of the web application.** A text describes the programming languages and the software programme used to create the application, its structure and design, and the pre-registration, registration and log-in functions. Click here for file

Additional file 4 **Technical details of acquisition of colposcopic images.** A text describes the types and technical specifications of the colposcopes, the video cameras, and the video editing softwares that were used for acquisition of images. Click here for file
