# Supplementary material for: Rationale and development of an on-line quality assurance programme for colposcopy in a population-based cervical screening setting in Italy
Source: BMC Health Serv Res. 2013 Jun 28;13:237. doi: 10.1186/1472-6963-13-237 (PMC3701540; doi:10.1186/1472-6963-13-237)
Supplement: Additional file 1 — Performance indicators routinely audited in colposcopy clinics of the Emilia-Romagna Region of Italy and observed data. A Table shows the list of performance indicators routinely audited in the colposcopy clinics of the Emilia-Romagna Region of Italy and, for each, the observed regional average and the range between health care districts. [file 1472-6963-13-237-S1.pdf]

Table Performance indicators routinely audited in colposcopy clinics of the Emilia-Romagna Region of Italy and observed data

No.	Definition	Regional average	Range between the 11 health care districts
1	Proportion of women with abnormal Pap smear results attending the clinic*	88%	69-100%
2	Proportion of women with Pap smear results of HSIL and carcinoma attending the clinic	91%	78-100%
3	Proportion of women with abnormal Pap smear results undergoing colposcopy within 4 weeks	58%	12-97%
4	Proportion of women with colposcopic findings interpreted as abnormal-grade 2 and suspected invasive cancer undergoing biopsy	95%	86-100%
5	Proportion of women with CIN3 and carcinoma and colposcopic findings interpreted as abnormal-grade 2 and suspected invasive cancer	65%	33-84%
6	Proportion of women with colposcopic findings interpreted as abnormal-grade 2 and suspected invasive cancer diagnosed with CIN3 and carcinoma	43%	33-58%
7	Proportion of women with Pap smear results of HSIL and carcinoma undergoing biopsy	92%	82-100%
8	Proportion of women with colposcopic findings interpreted as negative and partial or total invisibility of the SCJ	37%	5-64%
9	Proportion of women with histological diagnosis established before destructive therapy	90%	74-100%
10	Proportion of women with CIN biopsy and final histological diagnosis of invasive carcinoma	1%	0-4%

HSIL, high-grade squamous intraepithelial lesion; CIN3, cervical intraepithelial neoplasia grade 3; SCJ, squamocolumnar junction.

* Abnormal Pap smear results included the following: atypical squamous cells of undetermined significance; atypical squamous cells-cannot exclude high-grade lesion; atypical glandular cells; low-grade squamous intraepithelial lesion; HSIL; and carcinoma.

NOTE 1: The colposcopic categories referred to as negative, abnormal-grade 2, and suspected invasive cancer were equivalent to the categories of normal colposcopic findings, abnormal colposcopic findings-major changes, and colposcopic features suggestive of invasive cancer according to the International Federation for Cervical Pathology and Colposcopy classification of 2002 [Walker P, Dexeus S, De Palo G, Barrasso R, Campion M, Girardi F, Jakob C, Roy M: International terminology of colposcopy: an updated report from the International Federation for Cervical Pathology and Colposcopy. *Obstet Gynecol* 2003, 101:175-177].

NOTE 2: The data refer to the year 2010, when the colposcopy quality assurance programme was developed, with the exception of indicator No. 8 (year 2004). All indicators, part of which were taken from the guidelines set out for the NHS Cervical Screening Programme [Luesley D, Leeson S: *Colposcopy and programme management: guidelines for the NHS Cervical Screening Programme. Second edition.* Sheffield: NHS Cancer Screening Programmes; 2010], were calculated from cross-tabulated audit data collected by the regional Department of Health. National standards (according to the Italian Group for Cervical Cancer Screening, GISCi) are available only for indicators No. 1 (acceptable level ≥80%) and No. 2 (acceptable level ≥90%). The regional average level of quality of colposcopy service is generally acceptable, but with considerable variation between health care districts. This is especially the case for indicators No. 3, No. 5 (representing colposcopy sensitivity for CIN3 and carcinoma), and No. 8.
