# Supplementary material for: Rationale and development of an on-line quality assurance programme for colposcopy in a population-based cervical screening setting in Italy
Source: BMC Health Serv Res. 2013 Jun 28;13:237. doi: 10.1186/1472-6963-13-237 (PMC3701540; doi:10.1186/1472-6963-13-237)
Supplement: Additional file 2 — Digital colpophotographs representing the four categories of the International Federation for Cervical Pathology and Colposcopy classification of 2002. Four figures show digital colpophotographs taken with standard technique in one of the screening centres of the Emilia-Romagna Region of Italy, and considered by the programme Steering Committee to be well-representative of the definitions of normal colposcopic findings, abnormal colposcopic findings-minor changes, abnormal colposcopic findings-major changes, and colposcopic features suggestive of invasive cancer (in the programme, these categories were referred to as: negative; abnormal, grade 1; abnormal, grade 2; and suspected invasive cancer). [file 1472-6963-13-237-S2.pdf]

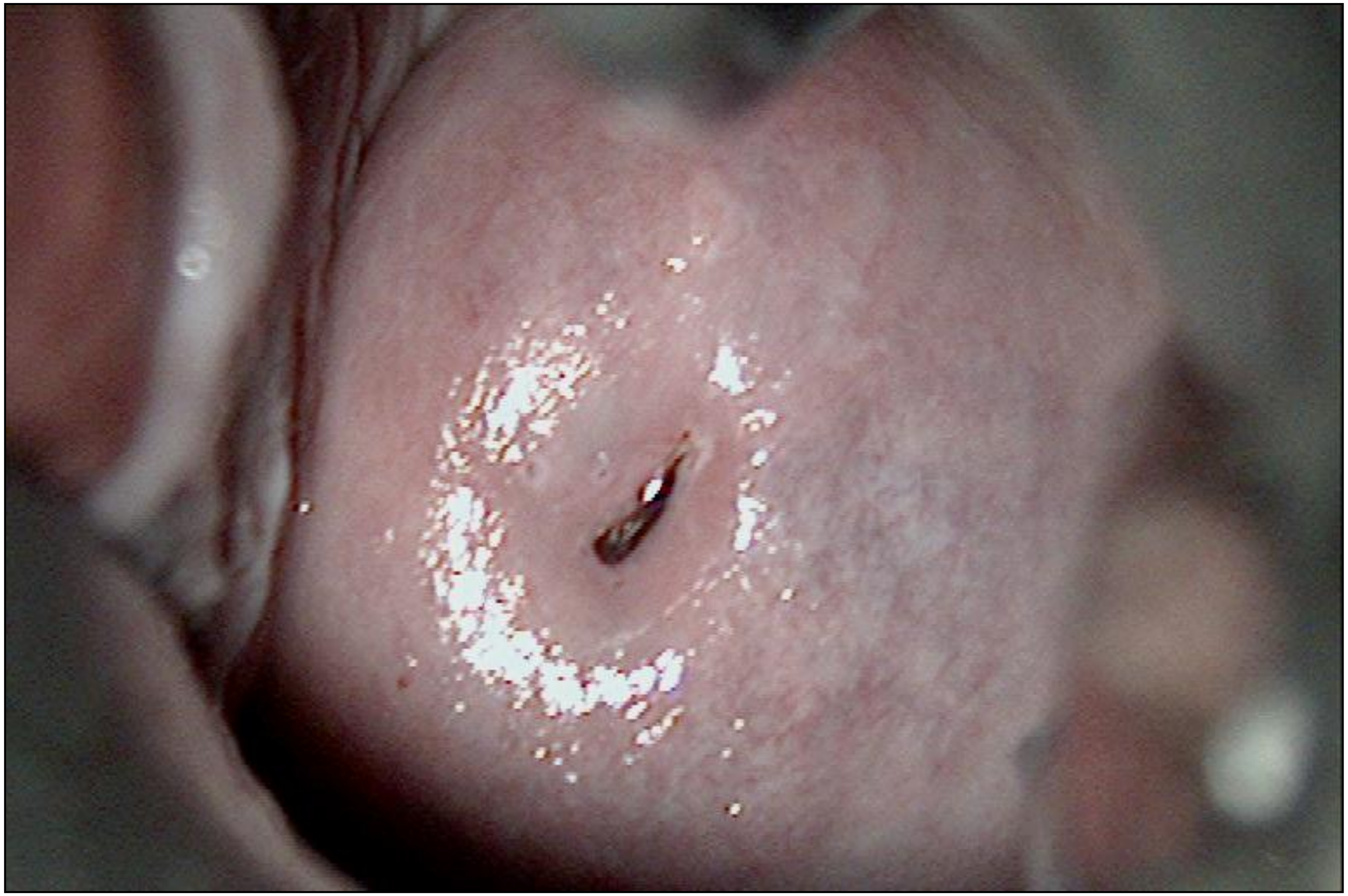

Digital colpophotographs representing the four categories of the International Federation for Cervical Pathology and Colposcopy classification of 2002

Figure 1.
Normal colposcopic findings (Negative)

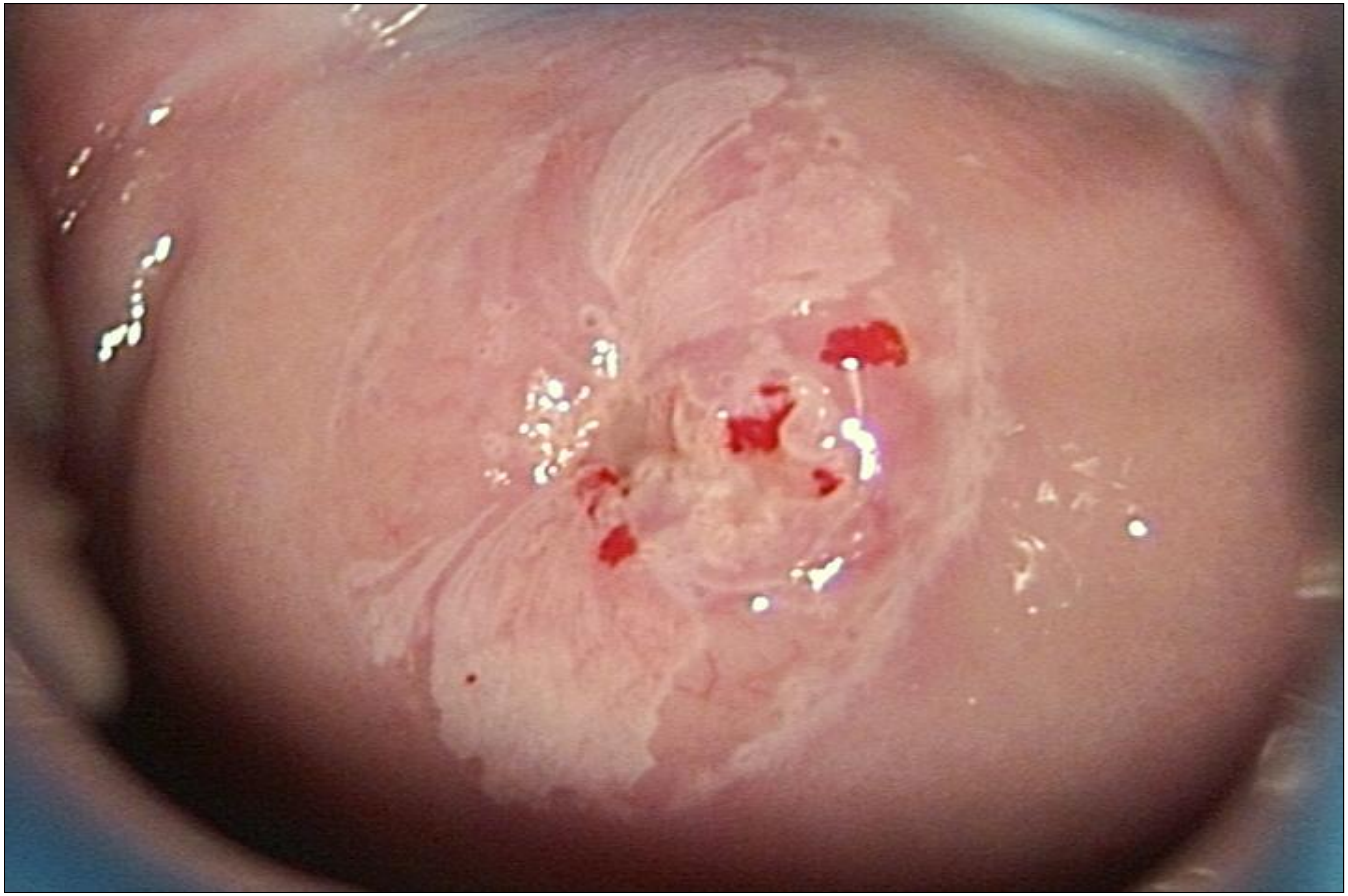

Digital colpophotographs representing the four categories of the International Federation for Cervical Pathology and Colposcopy classification of 2002

Figure 2.
Abnormal colposcopic findings-minor changes (Abnormal, grade 1)

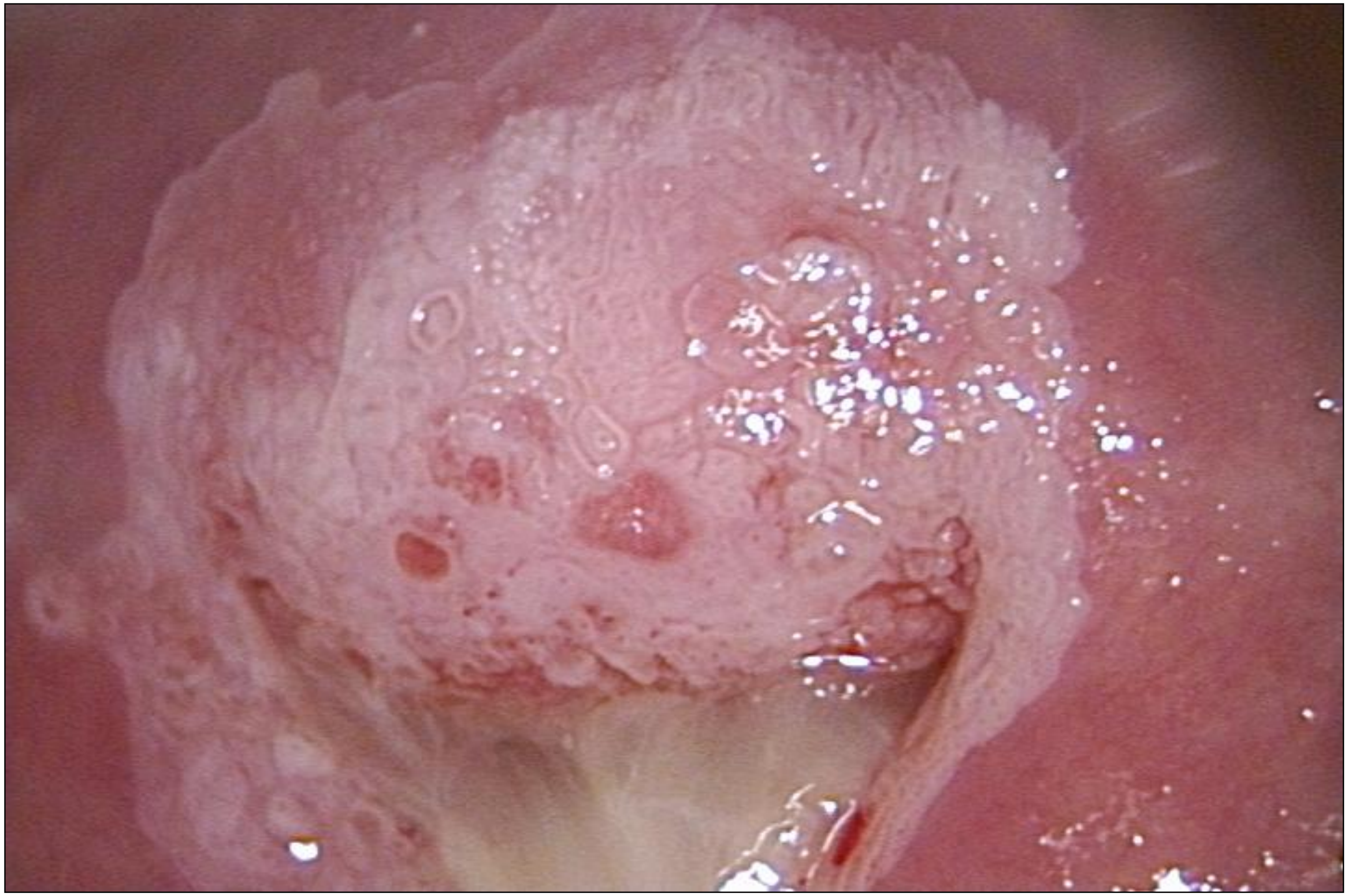

Digital colpophotographs representing the four categories of the International Federation for Cervical Pathology and Colposcopy classification of 2002

Figure 3.
Abnormal colposcopic findings-major changes (Abnormal, grade 2)

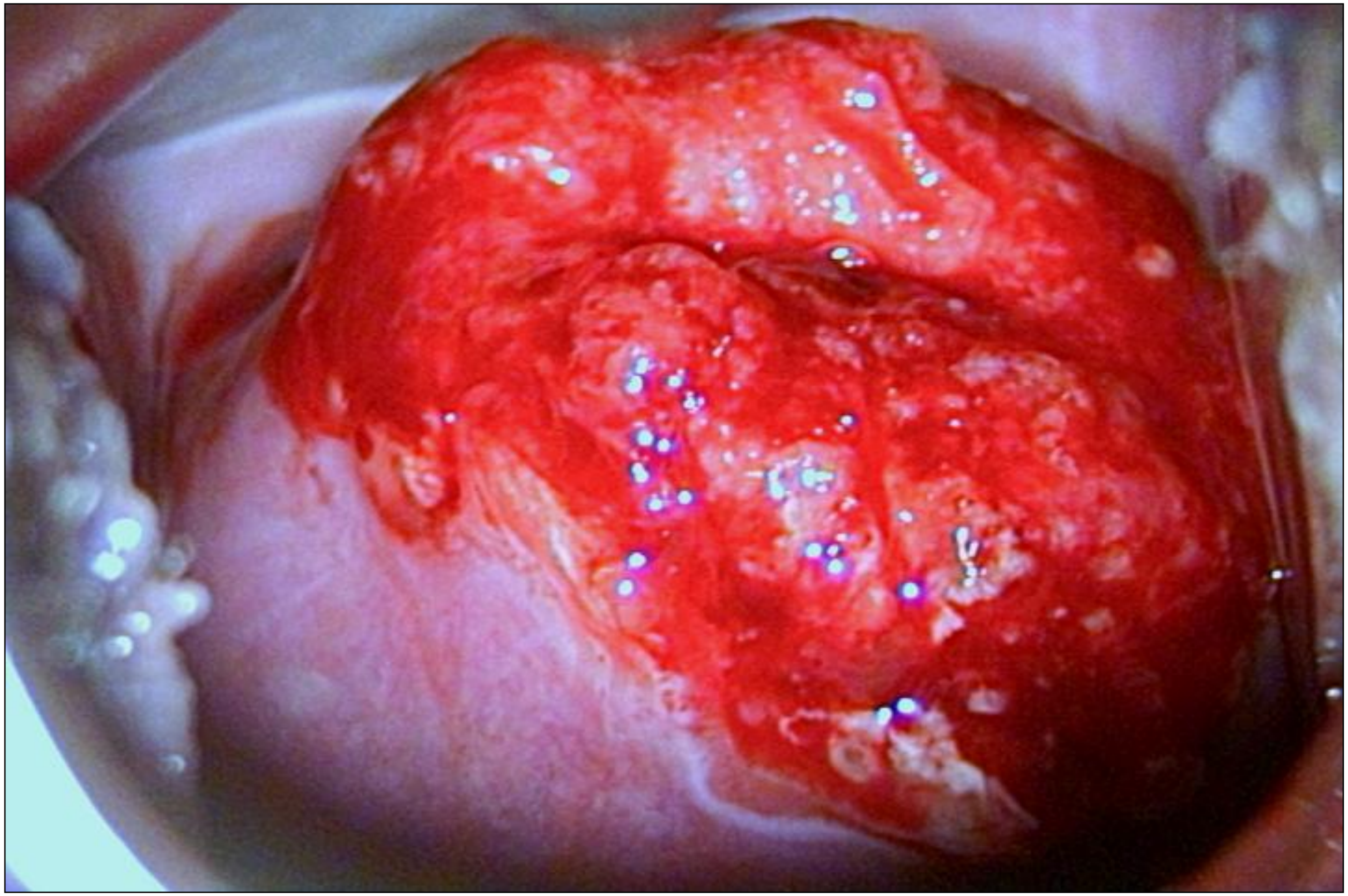

Digital colpophotographs representing the four categories of the International Federation for Cervical Pathology and Colposcopy classification of 2002

Figure 4.
Colposcopic features suggestive of invasive cancer (Suspected invasive cancer)
