# Supplementary material for: Rationale and development of an on-line quality assurance programme for colposcopy in a population-based cervical screening setting in Italy
Source: BMC Health Serv Res. 2013 Jun 28;13:237. doi: 10.1186/1472-6963-13-237 (PMC3701540; doi:10.1186/1472-6963-13-237)
Supplement: Additional file 3 — Technical details of the web application. A text describes the programming languages and the software programme used to create the application, its structure and design, and the pre-registration, registration and log-in functions. [file 1472-6963-13-237-S3.pdf]

Technical details of the web application

The web application in which the pre-registered users register, log-in, and access the test set of images through the Internet (https://sanita.regione.emilia-romagna.it/colposcopia) is the technical core of the programme. The application was developed as user-friendly as possible using Php and Javascript as programming languages and MySql as database system, and was mounted in a Linux server. All of the software is open-source.

(a) User pre-registration

Pre-registration at the coordinating centre is required for all users. The coordinating centre is based at the Cervical Cancer Screening Unit, Bologna Health Care District, Bologna, Italy, under the direction of Paolo Cristiani. The contact address for registration and inquiries is: Paolo Cristiani, Cervical Cancer Screening Unit, via Repubblica 11, San Lazzaro 40068, Bologna, Italy; telephone: +039 051 6224306/6224202; fax: +039 051 6224327; e-mail: p.cristiani@ausl.bologna.it.

For the first and any subsequent QA session targeting the staff of colposcopists working in the regional screening centres, potential participants should be preregistered by the directors of the district screening centres. This is aimed at identifying all target colposcopists. All other users should self-preregister, which involves giving the name and the identification code. The identification code consists of the fiscal code, an alphanumerical code composed by letters and numbers corresponding to name, sex, date and place of birth. Non-Italian visitors can access the web application and perform the test provided that they follow the preregistration and registration procedure. The only language of the application is Italian. The fiscal code of a non-Italian user can be created using a computer programme.

(b) User registration

The first step in utilization of the web application is the on-line registration, which permits each user to access his personal area. To register, the user's personal identification code must have been included beforehand in the application database. A personal password is chosen at the registration process, and the log-in access to the personal area is activated automatically. The user is asked to provide his e-mail address and some (non-obligatory) details of his professional experience and current activity. After the completion of the registration procedure, an e-mail notification will be delivered.

(c) Test section

Once the user is logged-in, a menu with a 'test section' and a 'scores section' is presented. In the test section, a test demo is available in order to familiarize with the procedure. Each of the 50 test cases has one high-definition digital image, a clinical history caption (patient age, last screening Pap smear result, previous cervical treatments, and HPV test result), and three multiple-choice questions. The most appropriate site for biopsy is indicated with a left-button mouse click on the chosen zone in the colposcopic image. The colposcopic image has the coordinates of the most appropriate site mapped inside the source code of the web application.

Each clinical case has to be completely answered before the user is enabled to pass to the next one. The user can stop the test and log-out from the web application at any moment. At the next log-in, the test will restart from the clinical case after the last one completed.

(d) Scores section

After finishing the test, in the scores section, the user will find a tabulation showing his classification of images according to colposcopic impression, visibility of the SCJ, and need for biopsy, and the classification given by the programme Committee. The biopsy site chosen by the user is expressed as correct/incorrect.
