# Supplementary material for: Rationale and development of an on-line quality assurance programme for colposcopy in a population-based cervical screening setting in Italy
Source: BMC Health Serv Res. 2013 Jun 28;13:237. doi: 10.1186/1472-6963-13-237 (PMC3701540; doi:10.1186/1472-6963-13-237)
Supplement: Additional file 4 — Technical details of acquisition of colposcopic images. A text describes the types and technical specifications of the colposcopes, the video cameras, and the video editing softwares that were used for acquisition of images. [file 1472-6963-13-237-S4.pdf]

Technical details of acquisition of colposcopic images

(a) Source

A basic set of 250 high-definition digital colpophotographs from the screening centres of the health care districts of Ravenna and Ferrara was selected. These centres had no qualitative peculiarities compared with the other screening centres in the region. The colpophotographs were taken during the routine colposcopic examination of two consecutive series of women with abnormal Pap smear results.

(b) Technical specifications

In the Ferrara centre, a KSK 150-FC binocular colposcope (Carl Zeiss Meditec AG, Jena, Germany) supplemented with a MediLive™ Trio Eye 3CCD video camera (Carl Zeiss Meditec AG, Jena, Germany), and a Pinnacle Studio 10.6 video editing software (Pinnacle Systems, Inc., Mountain View, CA) were used.

In the Ravenna centre, a KSK 150-FC binocular colposcope (Carl Zeiss Meditec AG, Jena, Germany), a JVC TK-1270 RGB CCD video camera (JVC Kenwood Electronics Italia, Milan, Italy), and a Colpox video editing software (Tesi Imaging, Pianiga, Venice, Italy) were used.

In both centres, the images were stored in the jpeg format.
